# Beyond Quadruple Therapy and Current Therapeutic Strategies in Heart Failure with Reduced Ejection Fraction: Medical Therapies with Potential to Become Part of the Therapeutic Armamentarium

**DOI:** 10.3390/ijms25063113

**Published:** 2024-03-07

**Authors:** Christos Kourek, Alexandros Briasoulis, Adamantia Papamichail, Andrew Xanthopoulos, Elias Tsougos, Dimitrios Farmakis, Ioannis Paraskevaidis

**Affiliations:** 1 Medical School of Athens, National and Kapodistrian University of Athens, 15772 Athens, Greece; chris.kourek.92@gmail.com (C.K.); alexbriasoulis@gmail.com (A.B.); adamantiapm@gmail.com (A.P.); 2 Department of Cardiology, University Hospital of Larissa, 41110 Larissa, Greece; andrewvxanth@gmail.com; 3 Department of Cardiology, Hygeia Hospital, 15123 Athens, Greece; cardio@tsougos.gr; 4 Attikon University Hospital, Medical School of Athens, National and Kapodistrian University of Athens, 12462 Athens, Greece; farmakis.dimitrios@ucy.ac.cy

**Keywords:** heart failure with reduced ejection fraction (HFrEF), angiotensin receptor/neprilysin inhibitors (ARNIs), sodium-glucose co-transporter-2 inhibitors (SGLT2), quadruple medical therapy, novel therapies, HeartMate 3 left ventricular assist device (LVAD)

## Abstract

Heart failure with reduced ejection fraction (HFrEF) is a complex clinical syndrome with significant morbidity and mortality and seems to be responsible for approximately 50% of heart failure cases and hospitalizations worldwide. First-line treatments of patients with HFrEF, according to the ESC and AHA guidelines, include β-blockers, angiotensin receptor/neprilysin inhibitors, sodium-glucose cotransporter 2 inhibitors, and mineralocorticoid receptor antagonists. This quadruple therapy should be initiated during hospital stay and uptitrated to maximum doses within 6 weeks after discharge according to large multicenter controlled trials. Quadruple therapy improves survival by approximately 8 years for a 55-year-old heart failure patient. Additional therapeutic strategies targeting other signaling pathways such as ivabradine, digoxin, and isosorbide dinitrate and hydralazine combination for African Americans, as well as adjunctive symptomatic therapies, seem to be necessary in the management of HFrEF. Although second-line medications have not achieved improvements in mortality, they seem to decrease heart failure hospitalizations. There are novel medical therapies including vericiguat, omecamtiv mecarbil, genetic and cellular therapies, and mitochondria-targeted therapies. Moreover, mitraclip for significant mitral valve regurgitation, ablation in specific atrial fibrillation cases, omecamtiv mecarbil are options under evaluation in clinical trials. Finally, the HeartMate 3 magnetically levitated centrifugal left ventricular assist device (LVAD) has extended 5-year survival for stage D HF patients who are candidates for an LVAD.

## 1. Introduction

Heart failure with reduced ejection fraction (HFrEF) represents a major clinical syndrome worldwide affecting around 1–3% of the general adult population of developed countries [1,2,3]. This prevalence increases accordingly with age, with individuals over 65–70 years old being affected in 6–10% of cases [1,2,3]. Despite the recent advances in medical therapies [4], HFrEF is still associated with increased morbidity and mortality which rises up to 50% after 5 years, leading to high rates of hospitalization [3].

The management of HFrEF has evolved considerably over the years, with a growing armamentarium of pharmacological and non-pharmacological therapies, aiming to improve outcomes in these patients [4]. Neurohormonal modulators such as angiotensin-converting enzyme inhibitors (ACEis), angiotensin receptor blockers (ARBs), beta blockers, and aldosterone receptor antagonists (MRAs) remain the key elements of treatment, while novel approaches such as angiotensin receptor/neprilysin inhibitors (ARNIs) and sodium-glucose co-transporter-2 inhibitors (SGLT2) seem to be quite promising therapeutic fields [5,6,7]. Overall, the above-mentioned advancements show great potential in the treatment of HFrEF patients, focusing on improvements in long-term outcomes and the enhancement of patient care. Device-based treatments, including implantable cardioverter-defibrillators (ICDs) and implantable cardiac resynchronization therapy (CRT) defibrillators, have shown enhancements in both mortality and cardiac function in selected HFrEF populations [5,6].

The aim of the present review is to provide a comprehensive aspect of the current guidelines regarding the treatment options in HFrEF and emerging novel therapeutic strategies.

## 2. Current Therapeutic Strategies in HFrEF

### 2.1. Medical Therapy

The 2021 ESC Guidelines for the Diagnosis and Treatment of Acute and Chronic Heart Failure [5], as well as the 2022 AHA/ACC/HFSA Guidelines for the Management of Heart Failure [6], proposed ARNIs, beta blockers, SGLT2 inhibitors, and MRAs as a class IA first line therapy in the treatment of HFrEF (Table 1). This quadruple therapy with β-blockers, ARNIs, SGLT2 inhibitors, and MRAs should be initiated during hospital stay and uptitrated to maximum doses within 6 weeks after discharge according to the STRONG HF trial [8], thus improving survival by approximately 8 years for a 55-year-old heart failure patient [9].

#### 2.1.1. Beta Blockers

The use of beta blockers in the management of HFrEF has been extensively studied. Their effect is attributed to their ability to antagonize the effects of catecholamines, leading to a reduction in HR, BP, and myocardial oxygen demand, as well as improvements in left ventricular (LV) function and remodeling [4]. Large-scale trials, such as the Carvedilol Prospective Randomized Cumulative Survival (COPERNICUS) trial [12], have shown that beta blockers, including carvedilol, reduce mortality and hospitalization rates. The COPERNICUS trial reported a 35% reduction in the risk of death and a 31% reduction in the risk of hospitalization for HF in patients treated with carvedilol compared to those receiving a placebo [12]. Similarly, the Metoprolol CR/XL Randomized Intervention Trial in Congestive Heart Failure (MERIT-HF) [13] demonstrated a 34% reduction in the risk of death and a 16% reduction in the risk of worsening HF symptoms in patients with HFrEF treated with metoprolol succinate compared to placebo. Additionally, the Cardiac Insufficiency Bisoprolol Study II (CIBIS-II) [14] showed a 34% reduction in the risk of death in patients with HFrEF treated with bisoprolol compared to placebo.

#### 2.1.2. Sodium Glucose Cotransporter 2 (SGLT2) Inhibitors

The use of SGLT2 inhibitors has emerged as a significant advancement in the management of HFrEF. According to recent research, SGLT2 inhibitors have shown promising outcomes in patients with HFrEF, irrespective of their diabetic status, age, LVEF, NYHA class, renal function, or co-morbidities [5,6]. The mechanisms by which SGLT2 inhibitors decrease the severity of heart failure are multifaceted. They include improved energy metabolism, increased natriuresis/diuresis, decreased inflammation, weight loss, improved cardiac remodeling, decreased hyperuricemia, and inhibition of the sympathetic nervous system, among others [15]. These mechanisms contribute to the overall positive impact of SGLT2 inhibitors on heart failure, making them a valuable addition to the armamentarium of HFrEF therapies [15]. All the updated guidelines have provided a class I recommendation for the use of empagliflozin and dapagliflozin to reduce the risk of HF hospitalization and death in all patients with symptomatic HF and LVEF ≤ 40% [5,6].

#### 2.1.3. Angiotensin-Converting Enzyme Inhibitors (ACEis)

ACEis have played a pivotal role in the management of HFrEF for many decades, demonstrating significant efficacy in improving patient outcomes. Both robust evidence from clinical trials and the current guidelines support their use in the management of HFrEF [5,6]. ACEis offer substantial mortality reduction, decreased HF-related hospitalizations, improved cardiac function, and a mitigated risk of disease progression [16,17]. Their benefits are attributed to their ability to inhibit the renin/angiotensin/aldosterone system (RAAS), leading to vasodilation, reduced sodium and water retention, as well as decreased cardiac remodeling [18]. These mechanisms contribute to the improvement in symptoms, exercise tolerance, and overall quality of life in patients [19,20]. Furthermore, ACEis have been shown to reduce the risk of sudden cardiac death (SCD) and the progression of HF [4].

Several clinical trials have demonstrated their significant benefits in improving the prognosis of patients with HFrEF. The SOLVD trial [21] showed a 16% reduction in mortality and a 26% reduction in the risk of hospitalization for HF in patients treated with enalapril compared to those receiving a placebo, while for the SAVE trial [22] the corresponding rates were 19% and 40%, accordingly, in patients treated with captopril compared to a placebo. The CONSENSUS trial [17] reported a 31% reduction in the risk of worsening HF symptoms with the use of enalapril.

#### 2.1.4. Angiotensin Receptor Blockers (ARBs)

ARBs also represent a cornerstone in the management of HFrEF, demonstrating substantial efficacy in improving patient outcomes. Their benefits are attributed to their ability to block the angiotensin II receptor, leading to the same positive effects as ACEis. Furthermore, they have been shown to reduce the risk of SCD and the progression of HF [23]. Their use has been suggested as an alternative to ACEis in patients with HFrEF who are intolerant to ACEis, with a class I, level of evidence A recommendation [5,6].

Clinical trials have consistently shown the mortality-reducing benefits of ARBs in HFrEF patients, with a significant reduction in the risk of all-cause mortality. Valsartan Heart Failure Trial (Val-HeFT) [24] demonstrated that valsartan reduced the combined risk of mortality and morbidity by 13% compared to placebo in HFrEF patients. Additionally, the CHARM-Alternative trial [25] showed that the use of candesartan reduced the risk of cardiovascular death or HF hospitalization by 23% compared to placebo in patients intolerant to ACEis.

#### 2.1.5. Mineralocorticoid Receptor Antagonists (MRAs)

MRAs have shown great effectiveness in managing HFrEF. The way MRAs affect cardiac function is by blocking mineralocorticoid receptors. This prevents effects caused by aldosterone, such as keeping sodium inside the body and taking potassium out of it, while also promoting fibrosis [26,27]. By changing these pathways, MRAs lessen the bad changes in heart structure and activation of neurohormones found in HFrEF.

Two randomized clinical trials, the RALES [28] and the EMPHASIS-HF trail [29], provided strong proof that using MRAs is helpful for people with HFrEF. The RALES study [28] showed that using spironolactone reduced the chance of mortality and hospitalizations due to HF by 30% and 35%, respectively, in people with severe HF. On the other hand, the EMPHASIS-HF trial [29] found a 37% reduction in the risk of cardiovascular death or HF hospitalization, with the use of eplerenone in patients with mild symptoms of HF. These results highlight how MRAs can greatly lower syndrome severity and mortality rates in patients with HFrEF. Finally, MRAs were shown to mitigate the progression of HFrEF [28,29].

#### 2.1.6. Diuretics

Diuretic therapy, including loop and thiazide diuretics, plays a crucial role in managing volume status in patients with HFrEF. While they do not provide mortality benefit, they are essential for treating congestion and managing fluid overload. Clinical trials and guidelines emphasize their importance in managing volume status in patients with HF. Loop diuretics such as bumetanide, ethacrynic acid, and furosemide are recommended for use when needed to manage volume status in patients with HF [5,6].

#### 2.1.7. Digoxin

The use of digoxin in the management of HFrEF has been the subject of extensive research in clinical trials. The 2021 ESC Guidelines for the diagnosis and treatment of acute and chronic heart failure recommend the use of digoxin as an additional or alternative therapy in patients with HFrEF, particularly in those with AF, with a class IIb, level of evidence B recommendation [5]. It has been shown to improve symptoms and reduce the risk of hospital readmissions at 30 days (HR, 0.74; 95% CI, 0.59–0.93), 1 year (HR, 0.81; 95% CI, 0.72–0.92), and 6 years (HR, 0.90; 95% CI 0.81–0.99) in HFrEF [30]. The mechanism of action of digoxin involves its ability to increase myocardial contractility and reduce HR, thereby improving cardiac output and symptoms in patients with HFrEF [31].

The Digitalis Investigation Group conducted a large, controlled trial of digoxin in patients with HF, which demonstrated that digoxin had no impact on mortality but decreased hospitalization rates [32]. It demonstrated that digoxin reduced the risk of hospitalization due to worsening HF by 28% and the combined risk of death or hospitalization by 6% in patients with HFrEF [32].

#### 2.1.8. Direct-Acting Vasodilators

The 2021 ESC Guidelines recommend the use of hydralazine and isosorbide dinitrate combination therapy as an additional or alternative therapy in patients with HFrEF, particularly in those who cannot tolerate ACE inhibitors or ARBs, with a class I, level of evidence A recommendation [5]. The mechanism of action of direct-acting vasodilators involves their ability to reduce afterload, improve ventricular/arterial coupling, and enhance cardiac output. These mechanisms contribute to the improvement in symptoms, exercise tolerance, and overall quality of life in patients with HFrEF. Furthermore, vasodilator therapy has been shown to reduce the risk of heart failure hospitalizations and improve long-term survival in patients with HFrEF [4,33].

The Vasodilator-Heart Failure Trial (V-HeFT) I and II trials [34,35] showed that the use of hydralazine and isosorbide dinitrate combination therapy reduced mortality by 25% and 16%, respectively, in patients with HFrEF compared to placebo. Additionally, the African American Heart Failure Trial showed a 43% reduction in the risk of death or hospitalization for HF in black patients with NYHA class III or IV HF who received a combination of isosorbide dinitrate and hydralazine compared to those who received placebo [36].

#### 2.1.9. Sinus Node Modulators

Ivabradine, a selective inhibitor of the If current in the sinoatrial node and a characteristic sinus node modulator, has demonstrated efficacy in the management of HFrEF. The mechanism of action of ivabradine involves reducing heart rate without affecting myocardial contractility or ventricular repolarization. By lowering heart rate, ivabradine reduces myocardial oxygen consumption and improves diastolic filling time, potentially leading to improved cardiac function and symptoms in patients with HFrEF [37]. The SHIFT trial [38] showed that ivabradine reduced the risk of cardiovascular death or hospitalization for worsening heart failure by 18% compared to placebo in patients with HFrEF and heart rates ≥ 70 beats per minute, with an NNT of 26. The use of sinus node modulators in HFrEF management is recommended for patients with sinus rhythm, heart rates ≥ 70 beats per minute, and who are taking the tolerated or target dosage of a beta blocker [5,6].

### 2.2. Device Therapy

Device therapies are based on the evaluation of the need for ICD placement for primary prevention in patients with LVEF ≤ 35% in order to prevent sudden cardiac death and based on the consideration of CRT in patients with evidence of ventricular desynchrony to improve symptoms and reduce hospitalizations.

#### 2.2.1. Implantable Cardioverter-Defibrillators (ICDs)

ICDs have been shown to lower mortality rates in certain patients with HFrEF. The MADIT II study [39] found a 31% decrease in all types of deaths when patients with past MI and LVEF below 30% received an ICD compared to those who did not. The Sudden Cardiac Death in Heart Failure Trial (SCD-HeFT) [40] found that in patients with NYHA class II or III chronic HF and LVEF of 35 percent or less, amiodarone has no favorable effect on survival, whereas ICD therapy reduces overall mortality by 23% (0.77; 97.5 percent confidence interval, 0.62 to 0.96; *p* = 0.007). Implantation of ICD has specific indications in HFrEF. It is usually suggested in a patient with previous MI and an LVEF of 35% or lower [5,6].

#### 2.2.2. Implantable Cardiac Resynchronization Therapy (CRT) Defibrillators

Cardiac resynchronization therapy (CRT) consists of double-chamber heart devices (biventricular pacemakers) with beneficial effects in patients with heart failure and desynchrony of the two ventricles. The Multicenter InSync Randomized Clinical Evaluation (MIRACLE) trial [41] showed that cardiac resynchronization results in significant clinical improvement in patients who have moderate-to-severe HF and an intraventricular conduction delay. Specifically, as compared with the control group, patients assigned to cardiac resynchronization experienced an improvement in the 6MWT (+39 vs. +10 m, *p* = 0.005), functional class (*p* < 0.001), quality of life (−18.0 vs. −9.0 points, *p* = 0.001), time on the treadmill during exercise testing (+81 vs. +19 s, *p* = 0.001), and ejection fraction (+4.6 percent vs. −0.2 percent, *p* < 0.001), while fewer patients in the group assigned to cardiac resynchronization required hospitalization (8 percent vs. 15 percent) or intravenous medications (7 percent vs. 15 percent) for the treatment of heart failure (*p* < 0.05 for both comparisons) compared to the controls.

Additionally, the Comparison of Medical Therapy, Pacing, and Defibrillation in Heart Failure (COMPANION) study [42] showed that patients with HF NYHA III or IV and a QRS duration ≥ 120 ms could reduce their risk of mortality or hospitalization by 34% with the use of CRT along with an electric shock device compared to pharmaceutical treatment alone. Biventricular pacemakers are recommended for patients with NYHA III or IV class HF, LVEF ≤ 35%, and QRS duration ≥ 150 ms, as well as for those with NYHA class II HF and LVEF ≤ 30% who remain symptomatic despite optimal medical therapy [5,6,42]. The choice of whether someone gets a biventricular pacemaker or not should always be individualized for each patient. Its appropriate use could be a very important tool for the management of patients with HFrEF.

## 3. Future Therapeutic Perspectives in HFrEF

Additional therapeutic strategies targeting other signaling pathways, as well as adjunctive symptomatic therapies, seem to be necessary in the management of HFrEF. Although second line medications have not achieved improvements in mortality, they seem to decrease heart failure hospitalizations [43]. Vericiguat, mitraclip for significant mitral valve regurgitation [44], ablation in specific atrial fibrillation cases [45,46], and omecamtiv mecarbil [47] are options under evaluation in clinical trials. Finally, HeartMate 3 magnetically levitated centrifugal LVAD has extended 5-year survival for stage D HF patients who are candidates for an LVAD [48].

### 3.1. Medical Therapy

#### 3.1.1. Vericiguat

Vericiguat, an oral soluble guanylate cyclase stimulator, has emerged as a potential therapeutic option in the management of HFrEF. The VICTORIA trial [49] evaluated the efficacy and safety of vericiguat in 5050 high-risk patients with chronic symptomatic HFrEF (EF < 45%) who had recently experienced decompensated HF. The primary outcome of the trial was a composite of death from cardiovascular causes or first HF hospitalization. The results demonstrated that vericiguat, when titrated to 10 mg daily, led to a significant 10% reduction in the primary endpoint, primarily driven by a reduction in HF hospitalization. Notably, the study population had a high-risk profile, and clinical events occurred early, leading to the pre-defined number of endpoint events being reached after a mean patient follow-up period of only 10.8 months. Despite this, the reduction in the primary endpoint was statistically significant. Furthermore, subgroup analysis of the VICTORIA [49] results revealed that vericiguat did not reduce the primary endpoint in patients with the highest quartile of entry NT-proBNP levels (>5314 pg/mL). However, it was associated with a 23% risk reduction in patients with NT-proBNP levels ≤ 4000 pg/mL, suggesting that the clinical course of patients with severe HF, as indicated by extremely high levels of natriuretic peptides, may not be improved by vericiguat [49]. The safety profile of vericiguat was also assessed in the VICTORIA trial [49]. Anemia was more common in patients who received vericiguat, while hypotension was equally common in both study groups. Importantly, there were no significant differences between groups in the rates of symptomatic hypotension or syncope, indicating that vericiguat could be administered safely when gradually uptitrated. In summary, the VICTORIA trial [49] demonstrated that vericiguat, as an adjunct to standard medical therapy, significantly reduced the risk of cardiovascular mortality and HF hospitalization in high-risk patients with chronic symptomatic HFrEF. These findings position vericiguat as a promising therapeutic option for the management of HFrEF, particularly in patients with NT-proBNP levels ≤ 4000 pg/mL [4].

#### 3.1.2. Omecamtiv Mecarbil

Omecamtiv mecarbil, a cardiac myosin activator, is a new drug option that enhances cardiac conditions in patients with underlying HFrEF by directly augmenting cardiac sarcomere function. Omecamtiv mecarbil augments cardiac contractility by selectively binding to the catalytic S1 domain of cardiac myosin, thus increasing the number of force generators (myosin heads) that can bind to the actin filament and initiate a power stroke at the start of systole [50,51]. This agent has demonstrated benefit in reducing heart rate, peripheral vascular resistance, mean left arterial pressure, and left ventricular end-diastolic pressure in animal models [52]. Additionally, it increases stroke volume, systolic ejection time and cardiac output, improves systolic wall thickening and ventricular remodeling, and decreases natriuretic peptide concentrations in patients with HFrEF [53,54,55]. Finally, it increases systolic ejection time (SET), cardiac myocyte fractional shortening without a significant increase in LV dP/dtmax, myocardial oxygen consumption, and myocyte intracellular calcium [55]. Omecamtiv mecarbil differs from other HF therapies because it directly targets systolic performance rather than modulating associated neurohormonal perturbations. Unlike other HFrEF treatments, omecamtiv mecarbil does not lower blood pressure, affect kidney function, or alter potassium homeostasis, allowing its use in patients with cardiorenal limitations that prevent the use of other HF therapies [56].

The GALACTIC-HF trial [47], a large randomized, double-blind, placebo-controlled study, evaluated the efficacy and safety of omecamtiv mecarbil in patients with HFrEF. The trial showed that omecamtiv mecarbil did not note a significant reduction in the primary goal, cardiovascular death or HF events, when compared to placebo. However, a pre-specified analysis showed that omecamtiv mecarbil modestly reduced the combined rate of cardiovascular death and hospitalization or other urgent treatment for heart failure over a median of 21.8 months (37.0% vs. 39.1% with placebo, HR 0.92, 95% CI 0.86–0.99) [47]. Also, the treatment was associated with a lower chance of dying from cardiac issues or HF events in patients who had higher baseline NT-proBNP levels [47]. The safety record of omecamtiv mecarbil is usually the same as that of other HF treatments. There were no major differences in bad side effects between it and a placebo. The safety record of omecamtiv mecarbil is usually the same as for other HF treatments. There were no major differences in bad side effects between it and a placebo. This study showed that omecamtiv mecarbil could help to lower HFHF in selected groups of HFrEF patients [47].

#### 3.1.3. Genetic and Cellular Therapies

Gene and cellular therapies in HFrEF have gained more space in the therapeutic armamentarium with promising opportunities. Gene therapy via the delivery of DNA or RNA material into the body aims to alter cardiac function and structure. Importantly, early studies have tried to use genes delivered by vectors—viruses like adenoviruses or adeno-associated viruses (AAVs). The latter introduce genetic molecules either into the main blood stream, or into the coronary flow [4].

A study delivered patients with the acquired form of HFrEF, a human SERCA2a gene, to their myocardium using an AAV type 1 serotype vector. At first, studies on single cells and tests with animals showed good results. However, a key study called CUPID-2 [57] used this method but it did not show any benefits in real patients, terminating the program from its further continuation. On the other hand, treatments with mesenchymal precursor cells (MPCs) are showing promising signs of preventing irreversible heart and brain pathologies like MI or stroke. This treatment option works especially well for people who have significantly high levels of C-Reactive Protein (CRP). In the randomized, double-blind, controlled DREAM-HF study [58] in 537 HFrEF patients, the primary endpoint was time-to-recurrent events caused by decompensated HFrEF or successfully resuscitated symptomatic ventricular arrhythmias, while secondary endpoints included components of the primary endpoint, time-to-first terminal cardiac events, and all-cause death. The primary endpoint was similar between treatment groups (HR: 1.17; 95% CI: 0.81–1.69; *p* = 0.41) as were terminal cardiac events and secondary endpoints. MPCs (rexlemestrocel-L) strengthened heart function at 12 months, as measured by LVEF and decreased cardiovascular death, MI, or stroke over a mean follow-up of 30 months. Specifically, a single intra-myocardial injection of 150 million cells of rexlemestrocel-L i. improved LVEF from baseline to 12 months to a significantly greater extent than controls across all patients with available echocardiograms (*p* = 0.021), with maximal benefit seen in patients with active inflammation as measured by the presence of baseline hsCRP ≥2 mg/L (*p* = 0.008); ii. Reduced the risk of MI or stroke by 57% [HR 0.43; 95% CI (0.23, 0.78)] in all treated patients compared with controls; iii. reduced the risk of MI or stroke by 75% [HR 0.25; 95% CI (0.09, 0.68)] in patients with inflammation (baseline hsCRP ≥ 2 mg/L) compared with controls; iv. Reduced the risk for time-to-first MACE, defined as cardiovascular death, MI or stroke, by 28% [HR 0.72; 95% CI: (0.51, 1.03)] in all-treated patients compared with controls; and v. reduced the risk for time-to-first MACE by 37% [HF 0.63; 95% CI: (0.39, 1.02)] in patients with inflammation (baseline hsCRP ≥ 2 mg/L) compared with controls. However, it did not further reduce the frequency of recurrent hospitalizations for worsening HF symptoms when added to maximal standard-of-care medicines for heart failure. These early discoveries are quite promising, but it is important to remember that the area of gene and cell treatments for HFrEF is still growing. We need more studies so we can be sure about these early results and expand them in a wider manner. These treatments bring up some difficulties and good chances as well. They show we still need strict tests on patients in the clinic for long follow-up periods to completely understand how best to help people with HFrEF [4].

#### 3.1.4. Mitochondria-Targeted Therapies

Mitochondrial dysfunction is common in heart failure and seems to be an important therapeutical target for the improvement in cardiac function. Mitochondrial abnormalities include impaired mitochondrial electron transport chain activity, an increased formation of reactive oxygen species, shifted metabolic substrate utilization, aberrant mitochondrial dynamics, and altered ion homeostasis [59]. Depending on the pathophysiological mechanism, therapies could target (i) mitochondrial reactive oxygen species (MitoQ with antioxidative efficacy and elamipretide which target cardiolipin); (ii) cellular Ca^2+^ handling (CGP-37157 which is a selective inhibitor of the NCLX cariporide which reduces cytosolic Na^+^ by selectively inhibiting the sarcolemmal sodium/hydrogen exchanger (NHE) type 1, and SGLT2 inhibitors); and (iii) cardiac metabolism (inhibitors of fatty acid oxidation such as etomoxir, trimetazidine, malonyl-CoA decarboxylase inhibitors, and SGLT2 inhibitors) [60].

Several types of microRNAs have been suggested as potential therapeutic targets of mitochondrial dysfunction, as they have been shown to inhibit mitochondrion-mediated apoptosis, improve cardiac function by regulating mitochondrial fission and fusion, and prevent pyroptosis [61,62,63,64]. However, these preliminary findings mainly derive from in vitro and preclinical animal models, and their potential for cardioprotection in humans needs to be evaluated through large clinical studies. Other promising therapies in mitochondrial dysfunction in HF are mitoTEMPOL, an mtROS scavenger that attenuates nicotine-induced myocardial remodeling and cardiac dysfunction [65], and coenzyme Q10, an electron carrier in mitochondria, with antioxidant activity that reduces all-cause mortality and hospitalization related to HF [66]. In addition, CGP-37157 is a selective inhibitor of the mitochondrial Na^+^/Ca^2+^ exchanger that was shown to maintain mitochondrial Ca2+ and ameliorate pathological myocardial remodeling, left ventricular dysfunction and arrhythmias [67]. Another promising therapy is perhexiline, which improves cardiac energetics and symptoms in HF by modifying the myocardial energy substrate through the inhibition of CPT [68,69]. Finally, the classic SGLT2 therapy improves mitochondrial energetics in the heart by offering β-hydroxybutyrate as an attractive substrate for oxidation and protection against HF [70], and NAD+ precursors may have a beneficial effect on HF by normalizing NAD+/NADH redox balance and thus improving myocardial energetics [71,72].

### 3.2. Interventional Therapeutic Strategies

#### 3.2.1. MitraClip in Severe Mitral Regurgitation

Consideration of MitraClip therapy as an established treatment option in patients with severe secondary mitral regurgitation is a significant aspect of HFrEF therapy. The COAPT study showed that among patients with HF and severe secondary mitral regurgitation who remained symptomatic despite maximal medical therapy, transcatheter mitral-valve repair resulted in a lower rate of hospitalization for HF by 47% and lower all-cause mortality within 24 months of follow-up by approximately 33% compared to medical therapy alone [73]. At a 5-year follow-up, in the same patients, MitraClip was safe and led to a lower rate of hospitalization and lower all-cause mortality than medical therapy alone [74].

#### 3.2.2. Ablation in Patients with Atrial Fibrillation

Ablation therapy has emerged as a promising intervention in the management of atrial fibrillation (AF) in patients with HFrEF. AF often exacerbates the existing cardiac dysfunction, leading to increased morbidity and mortality [75]. Ablation procedures aim to restore and maintain normal sinus rhythm by targeting the irregular electrical pathways in the atria responsible for AF. The rationale behind ablation in heart failure lies in its potential to improve left ventricular function, reduce symptoms, and enhance the overall quality of life for affected individuals [75,76,77,78,79]. By eliminating or modifying the abnormal electrical circuits contributing to AF, ablation may mitigate the detrimental effects of AF on cardiac hemodynamics and consequently alleviate heart failure symptoms. This therapeutic approach represents a valuable strategy in the multidisciplinary management of patients with concurrent AF and HFrEF, offering a potential avenue for improved clinical outcomes and enhanced quality of life [75,76,77,78,79].

The CASTLE HTx trial showed that in patients with symptomatic AF and end-stage HF, catheter ablation was associated with a decrease in the composite outcome of all-cause mortality, LVAD implantation, and urgent transplantation compared with medical therapy alone [46].

#### 3.2.3. HeartMate 3 Magnetically Levitated Centrifugal LVAD

The HeartMate 3 is a state-of-the-art left ventricular assist device (LVAD) designed for patients with advanced HFrEF. Notably, the HeartMate 3 utilizes a magnetically levitated centrifugal pump, a groundbreaking technology that enhances durability and minimizes complications associated with mechanical circulatory support [80,81]. The magnetic levitation system eliminates the need for mechanical bearings, reducing friction and wear within the device, thereby enhancing its reliability and potentially extending its lifespan [80,81]. This innovative design contributes to a significant reduction in adverse events such as pump thrombosis, a complication often encountered in earlier generation LVADs, promoting improved patient outcomes [80,81].

The HeartMate 3 has demonstrated remarkable efficacy in improving hemodynamics and functional capacity in HFrEF patients. Its centrifugal flow pump design ensures a more physiological blood flow, minimizing shear stress and potential damage to blood components [80,81]. Moreover, the device allows for continuous and pulsatile flow options, offering flexibility in tailoring support based on individual patient needs. With its advanced technology and positive clinical outcomes, the HeartMate 3 represents a crucial advancement in the field of mechanical circulatory support, providing a viable option for those awaiting heart transplantation or as destination therapy for patients who are ineligible for transplant. The MOMENTUM 3–5-Year Outcomes trial showed that a centrifugal-flow LVAD was superior at improving survival compared with an axial-flow pump [48]. Specifically, among patients with advanced heart failure, the use of the HeartMate 3 centrifugal-flow pump was superior to the Heartmate II axial-flow pump at improving survival over 5 years, while median survival with the HeartMate 3 device exceeded 5 years [48]. This benefit was due to a lower incidence of death due to device thrombosis, stroke, or bleeding for the centrifugal-flow pump vs. the axial-flow pump [48]. Serious adverse events (any bleeding, any stroke, and suspected or confirmed pump thrombosis) were lower with the centrifugal-flow pump vs. the axial-flow pump [48].

## 4. Hemodialytic Treatment in HFrEF Patients with End-Stage Renal Disease

Patients with HFrEF may present a decline in renal function, frequently resulting in chronic kidney disease (CKD). These patients have increased rates of cardiovascular events, as well as high morbidity and mortality rates [82]. Uremic cardiomyopathy is a condition characterized by diastolic dysfunction [83] in association with progressive left ventricular hypertrophy (LVH) [84], myocardial fibrosis [85], and edema [86] in patients with CKD. Uremic cardiomyopathy is associated with worse prognosis [87].

An early start of hemodialysis, and particularly nonconventional hemodialysis such as frequent hemodialysis, may delay its progression and appears to have an advantage over conventional hemodialysis [88]. Nonconventional hemodialysis is when patients receive 1.5 to 2.5 h of dialysis 5 or 6 days per week (short daily or frequent hemodialysis), in comparison to 3 days per week with conventional hemodialysis, while an early start of hemodialysis is when it is applied before the start of the irreversible injury to the myocardium, such as LV dilatation, along with systolic dysfunction and diminished LVEF [88]. The major problem of dialyzed CKD patients is that they may present a parallel worsening of LV diastolic and right ventricular (RV) systolic function, accompanied by RV dilation and LVEF decrease, as shown by Arcari L et al. [89].

In conclusion, although kidney transplantation may be a high-risk surgical procedure for a New York Heart Association (NYHA) class III HFrEF patient with end-stage renal disease, it could be a suggested treatment for a patient who is able and hemodynamically stable to undergo the procedure that has been shown to reverse uremic cardiomyopathy and confer a significant survival advantage over hemodialysis [88]. There are also recent data from a single-center analysis of 103 HFrEF patients undergoing renal transplants where there was no significant association between pre-renal transplant EF and post-transplant all-cause mortality or adverse CV outcomes [90].

## 5. Limitations

Although the therapeutic approach of HFrEF has extensively been studied in the last few decades, there are still some major limitations in our current knowledge. Many studies have included only a small sample of patients, and the follow–up period was short. Also, the fact that GDMT is not being used enough or at a strong dose in people with HFrEF shows us how vital it is to study in more depth. Furthermore, while gene and cell therapies seem to be helpful in early tests on animals or humans, we need randomized controlled trials with larger samples of patients. This will aid the confirmation of the different medications’ efficacy, limiting, at the same time, their negative effects.

## 6. Conclusions

Beyond the established quadruple therapy in HFrEF, including ARNIs, beta blockers, SGLT2, and MRAs, there are novel therapeutic approaches, medical and interventional, which may be the key elements of the appropriate and improved management of HFrEF patents. Specifically, vericiguat, omecamtiv mecarbil, genetic and cellular therapies, mitochondria-targeted therapies, mitraclip for significant mitral valve regurgitation, ablation in specific atrial fibrillation cases, and omecamtiv mecarbil and HeartMate 3 magnetically levitated centrifugal LVAD for stage D HF patients are options under evaluation in clinical trials with promising results, widely expanding the therapeutic armamentarium of the clinical syndrome.

## Figures and Tables

**Table 1:** An overview of the standard and the novel therapeutic options in HFrEF, including medical and device therapy.

Standard Therapies	Mechanisms of Action	Clinical Evidence/Trials
Beta blockers	Antagonize the effects of catecholamines Reduce HR, BP, and myocardial oxygen demand Result in improvements in left ventricular (LV) function and remodeling	COPERNICUS: reduce mortality by 35% and hospitalization rates by 31% MERIT-HF: 34% reduction in the risk of mortality and a 16% reduction in the risk of HF progression CIBIS-II: 34% reduction in the risk of mortality
Sodium Glucose Cotransporter 2 (SGLT2) inhibitors	Improve energy metabolism Increase natriuresis/diuresis Decrease inflammation Result in weight loss Improve cardiac remodeling Decrease hyperuricemia Inhibit the sympathetic nervous system	Meta-analysis of DAPA-HF and EMPEROR-Reduced trials: 13% reduction in all-cause death and 14% reduction in cardiovascular death. There is a 26% relative reduction in the combined risk of cardiovascular death or first HF hospitalization and a 25% decrease in the composite of recurrent hospitalizations for HF or cardiovascular death.
Angiotensin-converting enzyme inhibitors (ACEis)	Inhibit of the renin/angiotensin/aldosterone system (RAAS) Lead to vasodilation Reduce sodium and water retention Decrease cardiac remodeling	SOLVD: 16% reduction in mortality and a 26% reduction in the risk of hospitalization for HF SAVE trial: 19% reduction in mortality and a 40% reduction in the risk of hospitalization for HF CONSENSUS: 31% reduction in the risk of worsening HF symptoms
Angiotensin receptor blockers (ARBs)	Block the angiotensin II receptor	Val-HeFT: reduced the combined risk of mortality and morbidity by 13% CHARM-Alternative: reduced the risk of cardiovascular death or HF-hospitalization by 23%
MRAs	Block mineralocorticoid receptors	RALES: reduced the chance of mortality and hospitalizations due to HF by 30% EMPHASIS-HF: reduced the chance of mortality and hospitalizations due to HF by 35%
Diuretics	Diminishing sodium reabsorption at different sites in the nephron, thereby increasing urinary sodium and water losses.	They do not provide mortality benefit; they are essential for treating congestion and managing fluid overload.
Implantable cardioverter-defibrillators (ICDs)	Primary prevention in patients with LVEF ≤ 35% or fatal arrhythmias	MADIT II: 31% decrease in all types of deaths when patients with past MI and LVEF below 30% SCD-HeFT: in patients with NYHA class II or III chronic HF and LVEF of 35 percent or less, amiodarone has no favorable effect on survival, whereas ICD therapy reduces overall mortality by 23%
Implantable cardiac resynchronization therapy (CRT) defibrillators	Primary prevention in patients with LVEF ≤ 35%, wide QRS and desynchrony of the two ventricles	MIRACLE: improvement in the 6MWT (+39 vs. +10 m, *p* = 0.005), functional class (*p* < 0.001), quality of life (−18.0 vs. −9.0 points, *p* = 0.001), time on the treadmill during exercise testing (+81 vs. +19 s, *p* = 0.001), and ejection fraction (+4.6 percent vs. −0.2 percent, *p* < 0.001), while fewer patients in the group assigned to cardiac resynchronization required hospitalization (8 percent vs. 15 percent) or intravenous medications (7 percent vs. 15 percent)
Digoxin	Increases myocardial contractility and reduces HR, thereby improving cardiac output and symptoms	Improves symptoms and reduces the risk of hospital readmissions at 30 days (HR, 0.74; 95% CI, 0.59–0.93), 1 year (HR, 0.81; 95% CI, 0.72–0.92), and 6 years (HR, 0.90; 95% CI 0.81–0.99) in HFrEF. Moreover, it reduces the risk of hospitalization due to worsening HF by 28% and the combined risk of death or hospitalization by 6%.
Direct-acting vasodilators (e.g., hydralazine and isosorbide dinitrate)	Reduce afterload Improve ventricular/arterial coupling Enhance cardiac output	V-HeFT I and II: the use of hydralazine and isosorbide dinitrate combination therapy reduced mortality by 25% and 16%, respectively African American Heart Failure Trial: 43% reduction in the risk of death or hospitalization for HF in black patients with NYHA class III or IV
Sinus node modulators (e.g., ivabradine)	Reduce heart rate without affecting myocardial contractility or ventricular repolarization. Reduce myocardial oxygen consumption Improve diastolic filling time, potentially leading to improved cardiac function and symptoms.	SHIFT: ivabradine reduced the risk of cardiovascular death or hospitalization for worsening heart failure by 18% compared to placebo in patients with HFrEF and heart rates ≥70 beats per minute.
**Novel Therapies**	**Mechanisms of Action**	**Clinical Evidence/Application**
Vericiguat	Stimulates the cyclic guanosine monophosphate (cGMP) pathway through direct and indirect stimulation of soluble guanylate cyclase (sGC).	VICTORIA: 10% reduction in HF hospitalizations and 23% risk reduction in patients with NT-proBNP levels ≤ 4000 pg/mL. No significant adverse events.
Omecamtiv Mecarbil	Selectively binds to cardiac myosin resulting in activation and increase in rate of ATP hydrolysis.	GALACTIC-HF: reduced the risk of HF events, including hospitalizations and urgent visits for HF, by 8% compared to placebo. Also, it was associated with a lower chance of HF mortality in patients with higher baseline NT-proBNP levels.
Genetic and cellular therapies	Delivery of DNA or RNA material into the body, aiming to alter cardiac function and structure.	CUPID-2: delivered a human SERCA2a gene to patient myocardium using an AAV type 1 serotype vector, but it did not show any benefits. DREAM-HF: mesenchymal precursor cells strengthened heart function at 12 months, as measured by LVEF and decreased cardiovascular death, MI, or stroke over a mean follow-up of 30 months.
Mitochondria-targeted therapies	Mitochondrial reactive oxygen species Cellular Ca^2+^ handling Cardiac metabolism	Preliminary findings mainly derive from in vitro and preclinical animal models.
MitraClip in severe mitral regurgitation	The MitraClip device utilizes a catheter-delivered clip rather than a suture to create a double orifice in an attempt to reduce the regurgitation jet in severe secondary mitral regurgitation.	COAPT: among patients with HF and severe secondary mitral regurgitation who remained symptomatic despite maximal medical therapy, transcatheter mitral-valve repair resulted in a lower rate of hospitalization for HF by 47% and lower all-cause mortality within 24 months of follow-up by approximately 33% than medical therapy alone.
Ablation in patients with atrial fibrillation	Ablation procedures aim to restore and maintain normal sinus rhythm by targeting the irregular electrical pathways in the atria responsible for atrial fibrillation.	CASTLE HTx: in patients with symptomatic AF and end-stage HF, catheter ablation was associated with a decrease in the composite outcome of all-cause mortality, LVAD implantation, and urgent transplantation compared with medical therapy alone.
HeartMate 3 magnetically levitated centrifugal LVAD	HeartMate 3 utilizes a magnetically levitated centrifugal pump that enhances durability and minimizes complications associated with mechanical circulatory support. Its centrifugal flow pump design ensures a more physiological blood flow, minimizing shear stress and potential damage to blood components. Moreover, the device allows for continuous and pulsatile flow options.	MOMENTUM 3–5-Year Outcomes: among patients with advanced heart failure, the use of the HeartMate 3 centrifugal-flow pump was superior to the Heartmate II axial-flow pump at improving survival over 5 years while median survival with the HeartMate 3 device exceeds 5 years. Also, there was a lower incidence of death due to device thrombosis, stroke, or bleeding and lower serious adverse events (any bleeding, any stroke, and suspected or confirmed pump thrombosis).
**Other Therapies**	**Mechanisms of Action**	**Clinical Evidence/Application**
Kidney transplantation	Decreases left ventricular hypertrophy, increases LVEF, and improves survival in end-stage renal disease patients.	LVH shown on echocardiography declined after the first post-transplant year from 67% to 37%, confirming the positive relationship between LVH and the elimination of uremia-related risk factors after successful renal transplantation [10]. Overall, 69.9% of 103 kidney transplant recipients with LVEF ≤ 40% and heart failure prior to transplant achieved LVEF ≥ 50% (normal LVEF) and had improved survival after transplantation [11].

## Abbreviations

AAVs	Adeno-Associated Viruses
ACC	American College of Cardiology
ACE	Angiotensin-Converting Enzyme
ACEis	Angiotensin-Converting Enzyme inhibitors
AF	Atrial Fibrillation
ARBs	Angiotensin Receptor Blockers
ARNI	Angiotensin Receptor Blocker/Neprilysin Inhibitors
BNP	B-type Natriuretic Peptide
BP	Blood Pressure
CAD	Coronary Artery Disease
CHF	Congestive Heart Failure
CRP	C-Reactive Protein
CRT	Cardiac Resynchronization Therapy
GDMT	Guideline-Directed Medical Therapy
HF	Heart Failure
HfrEF	Heart Failure with reduced Ejection Fraction
HR	Heart Rate
ICDs	Implantable Cardioverter-Defibrillators
LV	Left Ventricular
LVEF	Left Ventricular Ejection Fraction
MI	Myocardial Infarction
MPCs	Mesenchymal Precursor Cells
MRAs	Mineralocorticoid Receptor Antagonists
NNT	Number Needed to Treat
NT-proBNP	N-Terminal pro-B-type Natriuretic Peptide
NYHA	New York Heart Association
RAAS	Renin/Angiotensin/Aldosterone System
SCD	Sudden Cardiac Death
SGLT2i	The use of sodium glucose cotransporter 2 inhibitors
